# Comparative Effectiveness of Radiation Versus Radical Cystectomy for Localized Muscle-Invasive Bladder Cancer

**DOI:** 10.1016/j.adro.2022.101157

**Published:** 2022-12-27

**Authors:** Yoshiyuki Yamamoto, Atsunari Kawashima, Toshitaka Morishima, Toshihiro Uemura, Akinaru Yamamoto, Gaku Yamamichi, Eisuke Tomiyama, Makoto Matsushita, Taigo Kato, Koji Hatano, Isao Miyashiro, Norio Nonomura

**Affiliations:** a Department of Urology, Osaka University Graduate School of Medicine, Suita, Japan; b Department of Urology, Osaka International Cancer Institute, Osaka, Japan; c Cancer Control Center, Osaka International Cancer Institute, Osaka, Japan

## Abstract

**Purpose:**

Radical cystectomy (RC) with neoadjuvant chemotherapy is the most commonly recommended treatment for muscle-invasive bladder cancer (MIBC), yet RC with urinary diversion remains an invasive treatment. Although some patients with MIBC gain good cancer control with radiation therapy (RT), its effectiveness remains under discussion. Therefore, we aimed to reveal the effectiveness of RT compared with RC for MIBC.

**Methods and Materials:**

Using cancer registry and administrative data from 31 hospitals in our prefecture, we recruited patients with bladder cancer (BC) initially registered between January 2013 and December 2015. All patients received RC or RT, and none had metastases. Prognostic factors for overall survival (OS) were analyzed by Cox proportional hazards model and log-rank test. Propensity score matching between the RC and RT groups was performed to examine the association of each factor with OS.

**Results:**

Among the patients with BC, 241 received RC and 92 received RT. Median ages of the patients receiving RC and RT were 71.0 and 76.5 years, respectively. Five-year OS rates were 44.8% for patients receiving RC and 27.6% for patients receiving RT (*P* < .001). Multivariate analysis for OS showed that older age, poorer functional disability, clinical node positive, and pathology of nonurothelial carcinoma were significantly associated with worse prognosis. A propensity score-matching model identified 77 patients with RC and 77 with RT. In this arranged cohort, there were no significant differences in OS between the RC and RT groups (*P* = .982).

**Conclusions:**

Prognostic analysis with matched characteristics showed that patients with BC receiving RT were not significantly different from those receiving RC. These findings could contribute to proper treatment strategies for MIBC.

## Introduction

Patients with muscle-invasive bladder cancer (MIBC) have had a poor prognosis, with a median overall survival (OS) time of 5 to 6 years when they received platinum-based neoadjuvant chemotherapy followed by radical cystectomy (RC), which is now standard therapy.^1^, ^2^, ^3^, ^4^ Yet RC, which requires some urinary diversion, is a comparatively invasive treatment that frequently results in decreased postoperative quality of life. In contrast, radiation therapy (RT) does not require urinary diversion and does not change the body image of patients. Trimodality therapy (TMT) combines maximal transurethral resection of the bladder tumor, chemotherapy, and RT. The aim of TMT is to preserve the bladder and quality of life without compromising the oncological outcome. Recently, cancer control with TMT for MIBC has been reported favorably to be 70% to 88% with complete response^5^, ^6^, ^7^, ^8^ and to have rates of 5- and 10-year OS of 48% to 57% and 19% to 35%, respectively.^5^, ^6^, ^7^, ^8^, ^9^, ^10^, ^11^ As a result of the improvement in the prognosis of patients with MIBC with the use of TMT, RT with concurrent chemotherapy is a treatment option, especially for well-selected patients or those unfit for RC.^12^, ^13^, ^14^ Generally, 17% to 29% of patients receiving RT for MIBC eventually need salvage cystectomy after RT because of local recurrence or bladder shrinkage.^7^^,^^8^^,^^15^ Currently, there are no randomized prospective trials that compare the clinical outcome of RC with that of RT in patients with MIBC, primarily because of the difficulty in comparing both treatments as the result of their quite-different treatment approaches. Some retrospective reports, consisting of a single-center cohort,^10^^,^^16^ a national cancer database,^17^ and a systematic review,^18^ showed that the outcomes of RT were not inferior to those of RC in patients with MIBC, but a clear conclusion still has not been reached.

In this study, we aimed to reveal whether the effectiveness of RT for MIBC was inferior to that of RC. This study was analyzed in 2 parts. First, all cases were examined and compared according to type of treatments. Next, propensity score matching (PSM) was used to compare matched pairs of patients. Prognostic analysis of patients with matched characteristics revealed that the patients receiving RT were not significantly different from those receiving RC. This clinical information may aid clinicians in selecting proper treatment for MIBC, considering maintenance of patient quality of life and preference.

## Methods and Materials

### Study design

We performed a multicenter retrospective cohort study using a database that included patients with cancer. The database consists of record-linked clinical databased on a hospital-based cancer registry and administrative data collected for the development of the Diagnosis Procedure Combination/Per-Diem Payment System. The details of the database have been described in previous reports.^19^, ^20^, ^21^ In brief, this hospital-based cancer registry collected detailed information about the diagnosis of newly diagnosed cancer, including topographic and morphologic codes according to the *International Classification of Diseases for Oncology*, Third Edition and the tumor, node, metastasis (TNM) classification of cancer at the time of diagnosis according to the Seventh Edition of the Union for International Cancer Control system, as well as demographic information, between January 2013 and December 2015. Mortality data from the population-based cancer registry that obtains information on vital statuses of residents livening in our prefecture were incorporated. The administrative data contain inpatient clinical abstracts for hospitalization episodes as well as health service use claims. The database, developed in collaboration with the Council for Coordination of Accredited Cancer Hospitals in our prefecture, comprises the data that were provided from 31 hospitals in our prefecture on a voluntary basis. These hospitals were accredited as cancer hospitals by the national or prefectural government, covering approximately one-half of the newly diagnosed cancer patients residing in the study region.

This study was approved by the institutional review board of the Osaka International Cancer Institute (Approval No. 19143). The requirement for informed consent was waived because of the retrospective nature of the study.

**Figure 1:**
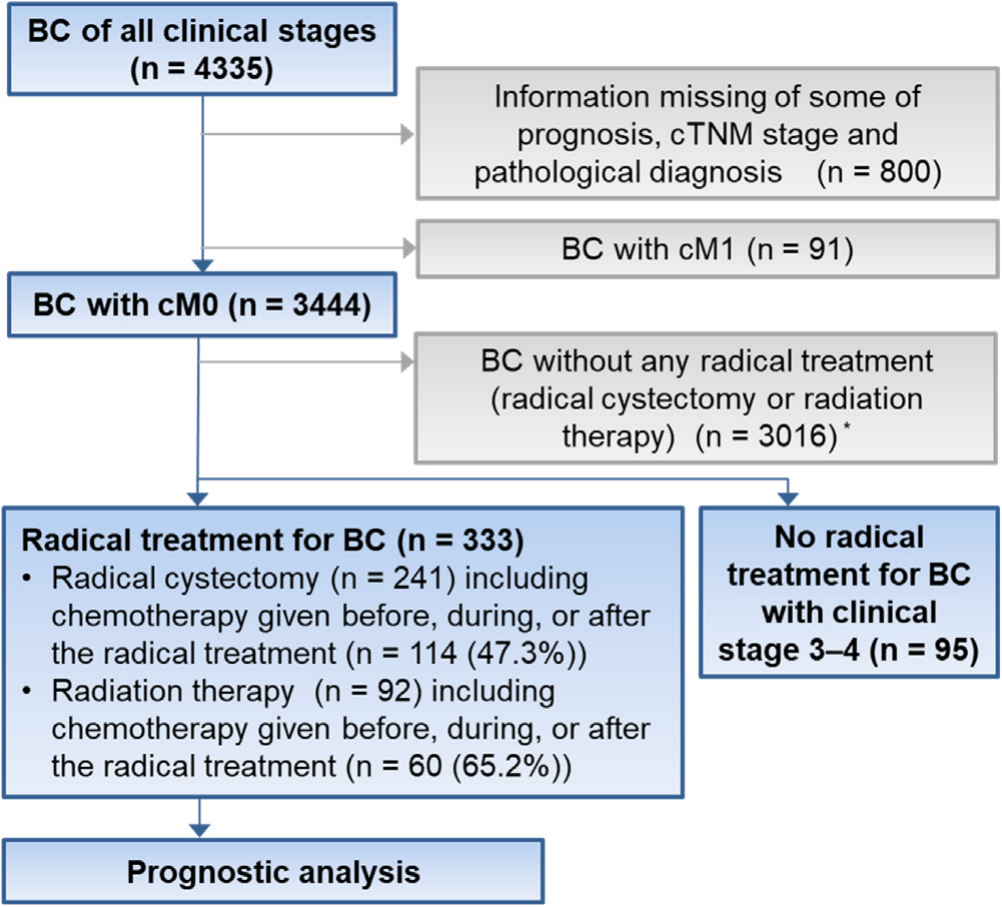
Study design and patient allocation. Blue rectangles and squares mean inclusion for analysis, and gray ones mean exclusion. *Patients with clinical stage 3 to 4 BC without any radical treatment for BC (n = 95) were not included. *Abbreviations:* BC = bladder cancer; cTNM = clinical tumor, node, metastasis.

We extracted information of patients who received a diagnosis of nonmetastatic bladder cancer (BC) at any of the 31 hospitals and who underwent RC or RT for BC (Fig. 1). Finally, we extracted 333 patients with BC, 241 who received RC and 92 who received RT. For the purpose of comparison between patients with BC with either RC or RT and those without radical treatments, we also extracted from the same database information of patients with nonmetastatic and clinical stage 3 to 4 BC who received no radical treatment. Five patients with both treatments were grouped into the initial treatment group. We collected clinical information that included age, sex, comorbidities, functional disability, pathology, clinical TNM stage, and the chemotherapy given before, during, or after the radical treatment from the database.

The degree of comorbidity per patient was measured using the updated score of the Charlson Comorbidity Index (CCI) according to diagnosis codes of the *International Classification of Diseases, Tenth Revision*.^22^^,^^23^ The score consisted of the sum of the individual component scores (ranging from 1-6) of 12 major illnesses (for example, heart failure and renal disease) that were associated with increased mortality. The score was categorized into no comorbidity (CCI score: 0), moderate comorbidities (1-2), and severe comorbidities (≥3).^24^ Next, the Barthel index was used to measure functional disability in activities of daily living (ADL) because of its use as a proxy of performance status in association with cancer survival.^24^ This index uses a scale of 0 to 100, with greater scores indicating better functional status, and patients were grouped into 4 categories: no disability (score: 100), moderate disability (60-99), severe disability (0-59), and unknown.^24^

The outcome of primary interest was OS. Patients without any radical treatments were excluded from the prognostic analysis. The duration of follow-up was defined as the period between the date of beginning cancer treatment and the date of last confirmation of survival or death from any cause. Patients were censored at the date of last follow-up with alive status from the registry data or the administrative data, whichever came later.

### Statistical analysis

Statistical analysis was performed using JMP Pro 15.0.0 (SAS Institute Inc, Cary, NC). Patient characteristics are presented as median ± range, and data were compared using the Wilcoxon test, Fisher exact test, and Pearson χ^2^ test. OS rates were calculated using the Kaplan–Meier method. Differences among different groups were assessed by log-rank test and were considered statistically significant when the *P* value was less than .05. Hazard ratios (HRs) for OS were calculated using the Cox proportional hazards model to check the relationship between survival and the predictive variables. Further, to ensure consistent distributions of clinical information for patients between the cystectomy group and RT group, the competing-risk regression model was adjusted by PSM. Through the process of patient matching, we matched each patient in one group to a possible patient in the other group, with all unmatched patients excluded from the PSM-adjusted competing-risk regression model. The variables for PSM were patients’ age, sex, CCI (no comorbidity vs moderate or severe comorbidities), Barthel index (no disability vs moderate vs severe), pathologic type (urothelial carcinoma [UC] vs non-UC), and clinical TN stage. The degree of each variable's difference between the RC and RT was assessed by standardized differences, which were calculated according to a previous report.^25^ Using the PSM-adjusted competing-risk regression model, the Kaplan–Meier method was conducted for some of the variables.

## Results

### Patient characteristics

We summarize the characteristics of the 333 patients, 241 receiving RC and 92 receiving RT, in Table 1. The median age of the patients undergoing RC and RT was 71 and 76.5 years (Range, 37-98 and 30-93 years), respectively (*P* < .001). The number of patients receiving RC with Barthel index scores of no/moderate/severe disability/unknown was 209 (86.7%), 22 (9.1%), 8 (3.3%), and 2 (0.8%), and those of the patients undergoing RT were 70 (76.1%), 7 (7.6%) 14 (15.2%), and 1 (1.1%), respectively (*P* < .001). Pathologic diagnosis showed that 233 (96.7%) patients in the RC group and 82 (89.1%) patients in the RT group had UC, and 8 (3.3%) and 14 (10.9%), respectively, received a diagnosis of non-UC (*P* = .012). Furthermore, the numbers of patients in the RC group with clinical node-negative and -positive status were 227 (94.2%) and 14 (5.8%), whereas those of the patients in the RT group were 76 (82.6%) and 16 (17.4%), respectively (*P* = .002). Chemotherapy given before, during, or after the radical treatment was received in some form by 114 (47.3%) of patients receiving RC versus 60 (65.2%) of patients receiving RT (*P* = .005). In the RC group, 79 (32.8%) patients received neoadjuvant chemotherapies, 215 (89.2%) underwent open RC, and 26 (10.8%) received laparoscopic surgery. A median of 30 (Range, 3-35) fractions of RT was administered. Salvage cystectomy after RT was performed in 3 (3.3%) patients with BC.

**Table 1:** Patient characteristics and outcomes

	Cystectomy (n = 241)		RT (n = 92)		*P*-value
Age, y, median, range	71	37-98	76.5	30-93	<.001
Sex					
Male	186	77.2%	64	69.6%	.159
Female	55	22.8%	28	30.4%	
Charlson Comorbidity Index					
0	141	58.5%	66	71.7%	.064
1-2	77	32.0%	18	19.6%	
>3	23	9.5%	8	8.7%	
Unknown	0	0.0%	0	0.0%	
Barthel index					
No disability	209	86.7%	70	76.1%	<.001
Moderate disability	22	9.1%	7	7.6%	
Severe disability	8	3.3%	14	15.2%	
Unknown	2	0.8%	1	1.1%	
Pathology					
UC	233	96.7%	82	89.1%	.012
Non-UC	8	3.3%	10	10.9%	
cT					
<1	62	25.7%	10	10.9%	.003
2	95	39.4%	34	37.0%	
>3	84	34.9%	48	52.2%	
cN					
0	227	94.2%	76	82.6%	
1	9	3.7%	11	12.0%	.004
2-3	5	2.1%	5	5.4%	
cM					
0	0	0.0%	0	0.0%	
Clinical stage					
<1	62	25.7%	10	10.9%	.002
2	91	37.8%	32	34.8%	
>3	88	36.5%	50	54.3%	
Chemotherapy around the radical treatment					
Yes	114	47.3%	60	65.2%	.005
No	127	52.7%	32	34.8%	
Duration of observation, y, median, range	3.29	0.03-6.27	1.78	0.15-6.29	.003
Outcome					
Alive	110	45.6%	26	32.1%	
Dead	131	54.4%	66	81.5%	

*Abbreviations:* cM = clinical M stage; cN = clinical N stage; cT = clinical T stage; RT = radiation therapy; UC = urothelial carcinoma.

We also compared the 333 patients who received any radical treatments (RC or RT) with 95 patients with clinical stage 3 to 4 and who received no radical therapies (Table E1). Patients without any radical treatments were significantly older and had poorer Barthel index scores than those receiving some treatments.

### OS and *risk factors*

**Figure 2:**
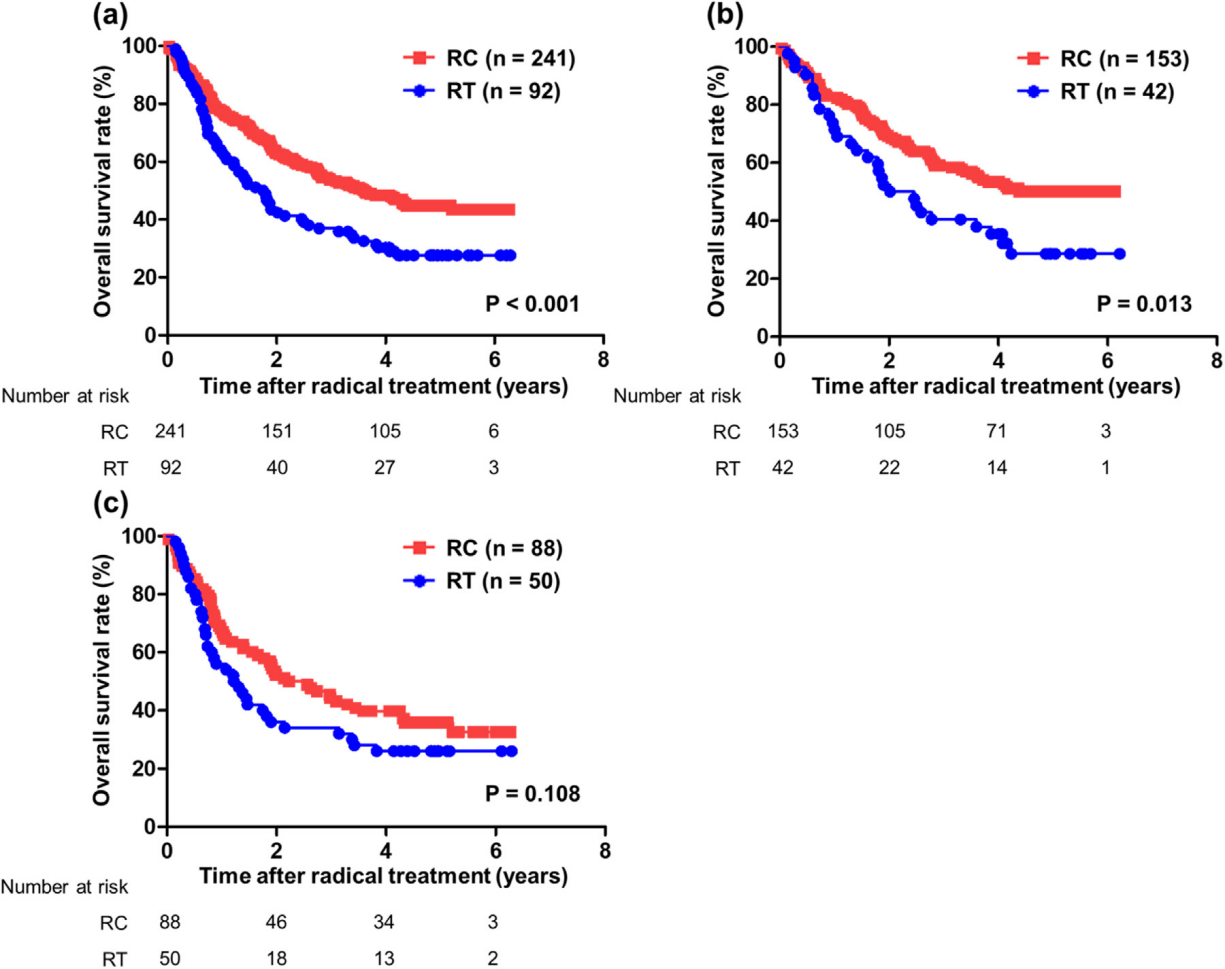
Prognostic analysis between the cystectomy group and radiation group for OS after radical treatment in 333 patients with BC. OS was analyzed by Kaplan–Meier analysis and log-rank test. (a) Patients with all clinical stages. (b) Patients with clinical stages less than 2. (c) Patients with clinical stages greater than 3. *Abbreviations:* BC = bladder cancer; OS = overall survival; RC = radical cystectomy; RT = radiation therapy.

**Figure 3:**
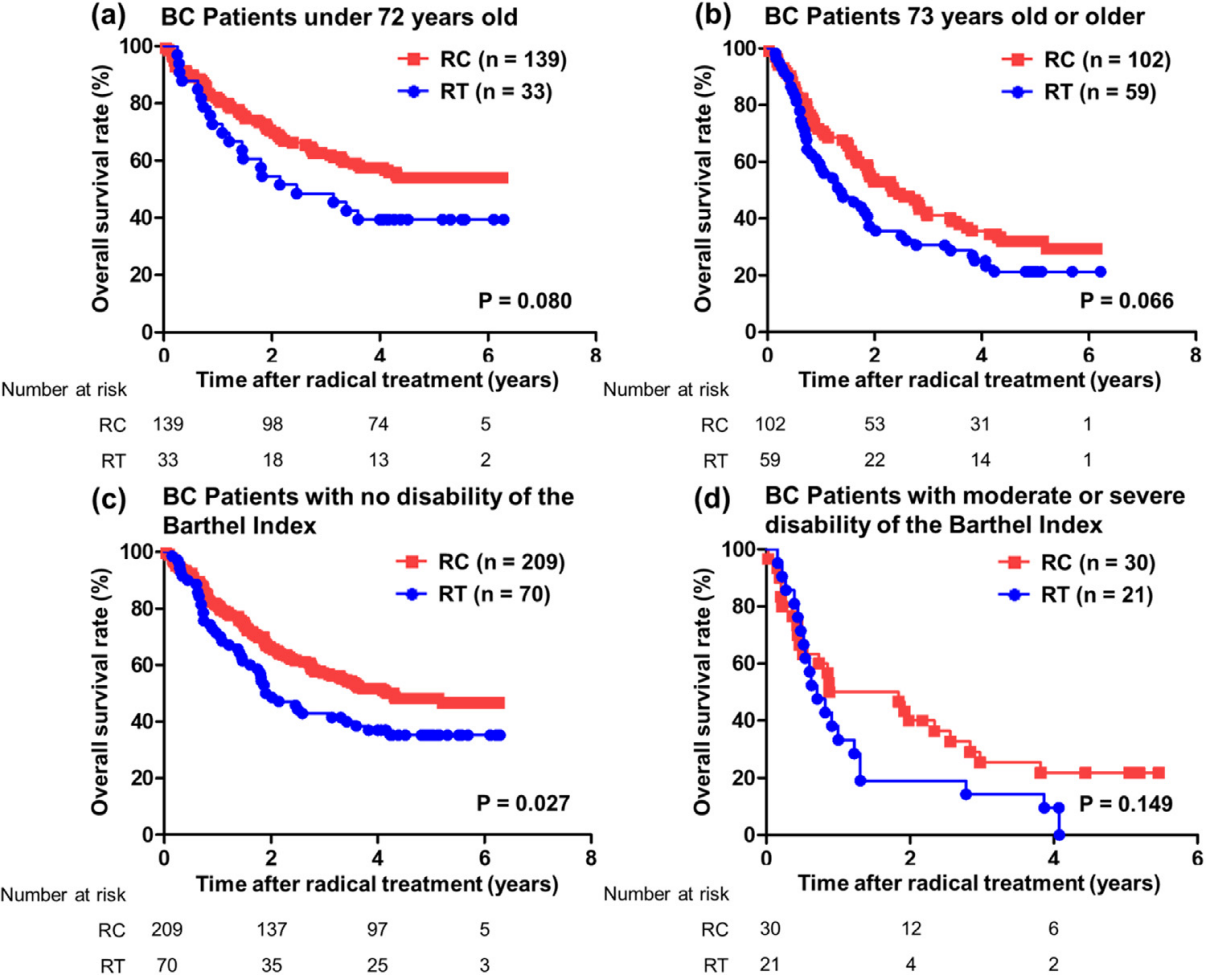
Prognostic analysis between the cystectomy group and radiation group for OS after radical treatment in 333 patients with BC. OS was analyzed by Kaplan–Meier analysis and log-rank test. (a) Patients younger than 72 years old (median value). (b) Patients 73 years old or older. (c) Patients with no disability as indicated by the Barthel Index. (d) Patients with moderate or severe disability. *Abbreviations:* BC = bladder cancer; OS = overall survival; RC = radical cystectomy; RT = radiation therapy.

At the time of analysis, 131 (54.4%) patients receiving RC and 66 (81.5%) patients receiving RT had died. The median OS times of the patients receiving RC and RT were 3.62 and 1.78 years, and rates of 5-year OS were 44.8% and 27.6%, respectively (*P* < .001) (Fig. 2a). Furthermore, the median OS time of the patients receiving RT with chemotherapy given before, during, or after the radical treatment was 1.84 years, and the difference between RT with some chemotherapy and RC both with and without chemotherapy had tendency (*P* = .062) (Fig. E1). The median OS times for the patients receiving RC and RT with clinical stage 2 or less were not reached and 2.24 years (*P* = .013) (Fig. 2b), whereas those with clinical stage 3 and more were 2.40 and 1.27 years, respectively (*P* = .108) (Fig. 2c). The association of prognosis with age showed a tendency for both younger and more elderly patients in RC group to have better OS than those in RT group (<72 years [median value], *P* = .080; >73 years, *P* = .066, respectively) (Fig. 3 a-b). For the association of prognosis with Barthel index, although patients with no disability had significantly better OS in RC versus RT groups (*P* = .027) (Fig. 3c), those with moderate and severe disability showed no differences of OS between RC and RT groups (*P* = .149) (Fig. 3d).

Next, multivariate analysis for OS showed that older age, poorer Barthel index, clinical node positive, and pathology of non-UC were significantly associated with worse prognosis (HR, 7.631, *P* < .001; HR, 3.222, *P* < .001 [severe disability vs no] and HR, 1.809, *P* = .012 [moderate disability vs no]; HR, 2.450, *P* < .001; and HR, 2.132, *P* = .009, respectively; Table 2). The patients with RT showed significantly poorer OS than those with RC only in the univariate analysis; there were no significant differences in the multivariate analysis (HR, 1.048, *P* = .784).

**Table 2:** Prognostic analysis of overall survival in all 333 patients with bladder cancer

	Univariate			Multivariate		
Characteristics	HR	95% CI	*P*-value	HR	95% CI	*P*-value
Age, range	16.239	5.589-48.453	<.001	7.631	2.524-24.077	<.001
Sex, male vs female	1.018	0.739-1.404	.911			
Charlson Comorbidity Index, >1 vs 0	1.091	0.819-1.454	.551			
Barthel index						
Severe disability vs no	3.787	2.364-6.066	<.001	3.222	1.909-5.438	<.001
Moderate disability vs no	2.037	1.313-3.162	.002	1.809	1.141-2.868	.012
cT, >3 vs <2	1.496	1.129-1.981	.005	1.311	0.968-1.776	.080
cN, 1-3 vs 0	2.427	1.590-3.704	<.001	2.450	1.554-3.865	<.001
Pathology, UC vs non-UC	1.999	1.180-3.387	.010	2.132	1.213-3.748	.009
Radical treatment, radiation vs cystectomy	1.645	1.223-2.214	.001	1.048	0.748-1.469	.784
Chemotherapy around the radical treatment	1.081	0.817-1.431	.587			

*Abbreviations:* BC = bladder cancer; CI = confidence interval; cN = clinical N stage; cT = clinical T stage; HR = hazard ratio; UC = urothelial carcinoma.

### OS analysis with a PSM-adjusted competing-risk regression model

**Figure 4:**
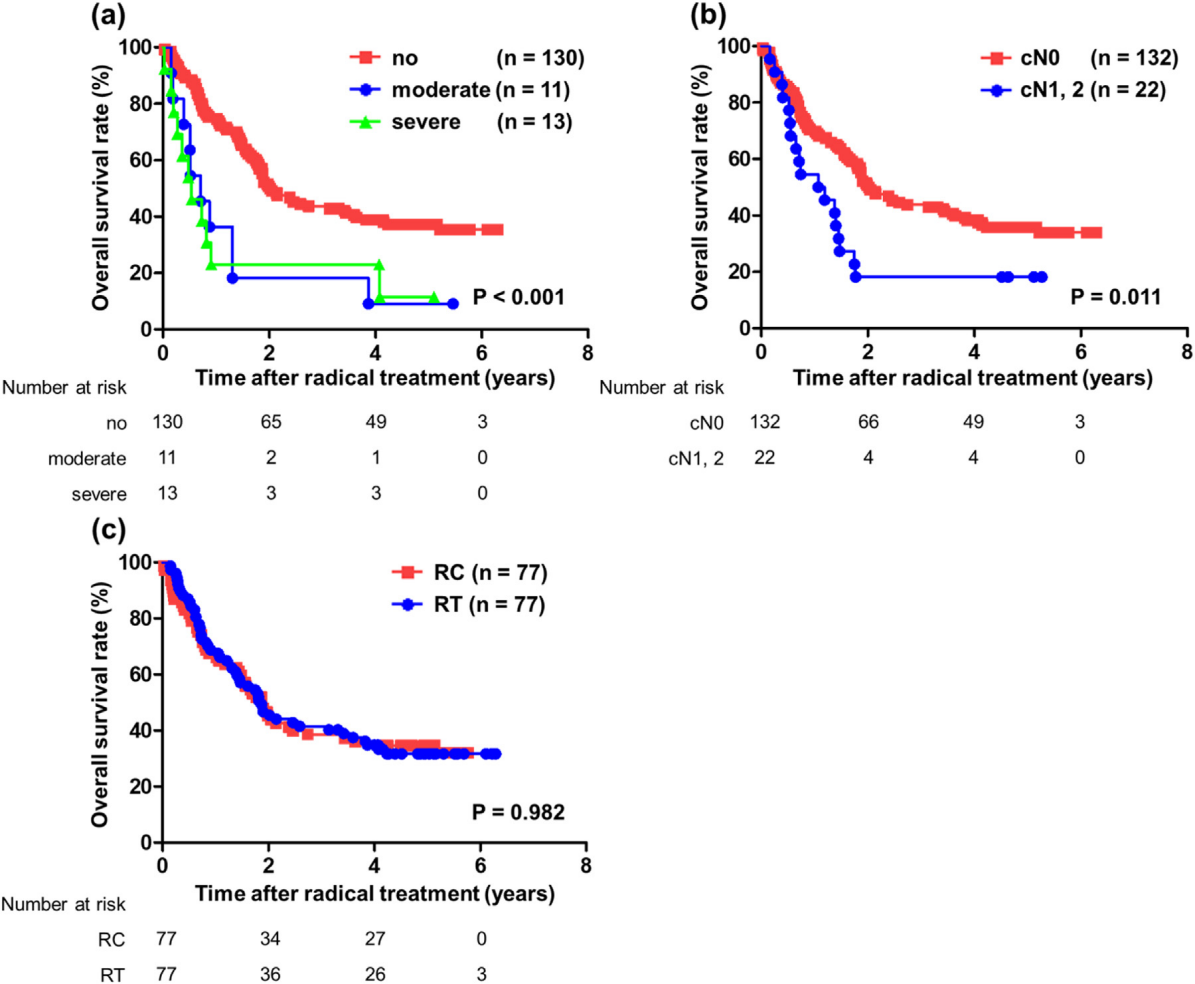
Prognostic analysis for OS after radical treatment in 154 patients with BC whose backgrounds were adjusted using propensity score matching. OS was analyzed by Kaplan–Meier analysis and log-rank test. (a) The association with functional disability as indicated by the Barthel Index. (b) The association with cN information. (c) The association with radical treatments (cystectomy group vs radiation group). *Abbreviations:* BC = bladder cancer; cN = clinical N stage; OS = overall survival; RC = radical cystectomy; RT = radiation therapy.

This cohort showed many apparent differences between the RC and RT groups, and analysis of OS by a PSM-adjusted competing-risk regression model for the exclusion of those differences was conducted. After PSM, the RC and RT groups each consisted of 77 patients (Table E2). In this model, poor ADL and clinical node positive were significantly associated with worse OS (no disability vs moderate vs severe, median survival 2.02 vs 0.71 vs 0.54 years, *P* < .001; cN0 vs cN1 and more, median survival 2.02 vs 1.13 years, *P* = .011, respectively; Fig. 4 a and b). However, there were no significant differences in OS between the patients receiving RC versus RT (median survival 1.90 vs 1.87 years, *P* = .982, Fig. 4c).

## Discussion

Currently, RC with neoadjuvant chemotherapy is the most commonly recommended treatment for MIBC or BC without metastases that is refractory to any treatment.^12^^,^^13^ Recently, the popularization of laparoscopic RC and robot-assisted RC (RARC) has been progressing worldwide, yet RC including open, laparoscopic, and RARC is still an invasive treatment and carries a high possibility of some complications.^26^^,^^27^ In the US and European guidelines, TMT with concurrent chemoradiation is recommended, especially for well-selected patients with MIBC or those unfit for RC.^12^, ^13^, ^14^ In the Asia consensus statements of National Comprehensive Cancer Network guidelines, RT could be considered as a therapeutic option for MIBC with cT2 as in Europe and the United States, yet perioperative treatments for bladder preservation vary considerably among each Asian countries.^28^ Overall, no successfully completed randomized controlled trials have compared the outcome of chemoradiation with RC. Thus, we evaluated the relative merits of RT compared with RC in a real-world setting.

In the prognosis of radical treatment for MIBC, chemoradiation therapy for MIBC enabled 70% to 80% of patients to attain a complete response with a 5-year rate of OS of approximately 50%.^5^, ^6^, ^7^, ^8^, ^9^^,^^11^ Chemoradiation has been shown to be superior to RT alone.^11^ Contrastingly, RC with neoadjuvant chemotherapy has enabled patients to achieve a 5-year rate of OS of 50% to 57%.^1^^,^^2^ Compared with the 5-year rates of OS in the previous reports,^5^, ^6^, ^7^, ^8^, ^9^^,^^11^ that for the RC group in the present study was almost equivalent (44.8%), but that for RT group was considerably worse (27.6%). This might be because, first, the patients receiving RT were comparatively older (Median, 76.5 years) and had a low rate of concurrent chemotherapy (65.2%). RT for MIBC often was used for patients with relatively older age or worse ADL, which corresponded to our result. Moreover, the patients receiving RT in the present study had significantly greater clinical T stage and clinical N stage stages than the patients receiving RC, so these backgrounds could have an effect on poor prognosis. Second, there were only a few patients receiving RT (3.3%) who underwent salvage cystectomy, which might be cause of disease progression and could also affect the poor outcome. Third, the present study duration was short, so if the study duration were extended, the OS for both groups might improve. Furthermore, the patients receiving RC had a worse prognosis after PSM (median OS time 3.62 years [before PSM] and 1.90 years [after PSM], respectively), because matching was performed with poorer prognostic characteristics.

In various clinical guidelines, RC generally was recommended first rather than RT, yet the present study could not reveal the superiority of RC to RT. Although the prognosis in the RT group was inferior to that in the RC group in all cohorts, the difference disappeared when the backgrounds of the clinical characteristics of both groups were matched. Previous retrospective reports similarly showed no significant differences of prognosis between the RC and RT groups,^10^^,^^16^^,^^17^ for which various reasons could be responsible. First, a comparatively small number of the patients receiving RC received neoadjuvant chemotherapy. Despite 74.3% of the patients receiving RT having a clinical stage greater than 2, only 32.8% patients received neoadjuvant chemotherapy. Second, only a few patients receiving RT underwent laparoscopic surgery, and moreover, no patients who underwent RT received RARC. Some reports have shown no prognostic superiority of RARC to open surgery.^29^^,^^30^ In contrast, opposite results of the superiority of RARC to open surgery have been reported,^31^ and the prognostic superiority of RARC to open surgery remains under discussion; thus, it is possible that RARC may contribute to better survival in patients with MIBC. If RARC can enable elderly patients and those with lower ADL to safely undergo radical surgery,^31^ additional comparative studies between RC and RT would be expected.

This study has some limitations. It was retrospective, based on registry data, and recruited patients with multiple clinical backgrounds from many hospitals. Thus, detailed pathologic and prognostic factors could not be analyzed that might affect patient prognosis in both groups. In both the RC and RT groups, the adaptations and regimens of chemotherapy around the radical treatment were diverse. There was no information about simultaneous lymphadenectomy in the patients receiving RC, whereas in the patients receiving RT, we could obtain no information about RT fields and doses. Although RT with chemotherapy generally is recommended for MIBC, 34.8% of patients with MIBC had RT without chemotherapy in this study. Further investigations are needed to validate our results in larger numbers of patients through multi-institutional prospective studies. This study revealed only that OS of the RT group was not inferior to that of the RC. However, RT for BC also carries some risk, including 20% to 30% chance or requirement for salvage cystectomy and dysuria related to a contracted bladder and pollakiuria, and the change in quality of life and patient satisfaction as a result of these risks were not analyzed. Additional studies will be needed to examine not only the change in quality of life and satisfaction after these treatments but also the survival results.

## Conclusions

Our prognostic analysis of patients with matched characteristics in the real world showed that outcomes for the patients receiving RT were not significantly different from those receiving RC. This clinical information might aid clinicians in selecting proper treatment for MIBC considering maintenance of patient quality of life and preference.

## Appendix Supplementary materials

Image, application 1

Image, application 2

Supplementary materials Supplementary material associated with this article can be found, in the online version, at XXX Image, application 3
