# Supplementary material for: Comparative Effectiveness of Radiation Versus Radical Cystectomy for Localized Muscle-Invasive Bladder Cancer
Source: Adv Radiat Oncol. 2022 Dec 27;8(3):101157. doi: 10.1016/j.adro.2022.101157 (PMC9991535; doi:10.1016/j.adro.2022.101157)
Supplement: Supplementary file 1 [file mmc1.docx]

# Supplemental figure 1


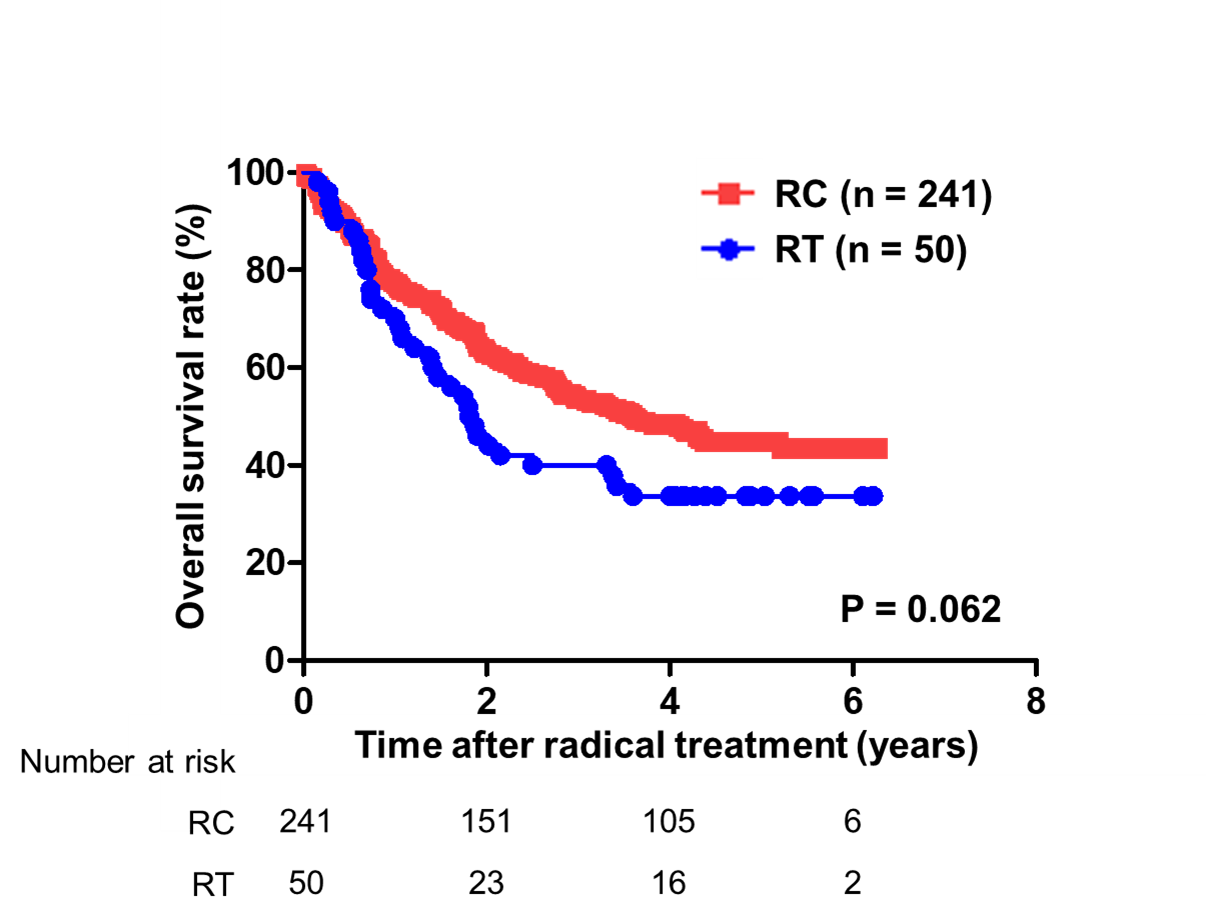


Supplemental figure 1. Prognostic analysis between BC patients receiving RT with some chemotherapy and RC both with and without chemotherapy for OS. Chemotherapy was given before, during, or after the radical treatment. OS was analyzed by Kaplan–Meier analysis and log-rank test. BC, bladder cancer; OS, overall survival; RC, radical cystectomy; RT, radiation therapy
