# Supplementary material for: Comparative Effectiveness of Radiation Versus Radical Cystectomy for Localized Muscle-Invasive Bladder Cancer
Source: Adv Radiat Oncol. 2022 Dec 27;8(3):101157. doi: 10.1016/j.adro.2022.101157 (PMC9991535; doi:10.1016/j.adro.2022.101157)
Supplement: Supplementary file 2 [file mmc2.docx]

| **Supplemental table 1** Comparison of characteristics of patients who had any radical treatments with those did no ones. | | | | | | | | | | |
| --- | --- | --- | --- | --- | --- | --- | --- | --- | --- | --- |
|  |  | Radical treatment | | | | | | | |  |
|  |  | Yes (n=333) | | |  | No (n=95) | | |  | P-value |
| Age: years, median (range) |  | 72 | ( | 30-98 | ) | 78 | ( | 45-96 | ) | <0.001 |
| Sex | Male | 250 | ( | 75.1% | ) | 69 | ( | 72.6% | ) | 0.689 |
|  | Female | 83 | ( | 24.9% | ) | 26 | ( | 27.4% | ) |  |
| Charlson Comorbidity Index | 0 | 207 | ( | 62.2% | ) | 59 | ( | 62.1% | ) | 0.587 |
|  | 1-2 | 95 | ( | 28.5% | ) | 21 | ( | 22.1% | ) |  |
|  | Over 3 | 31 | ( | 9.3% | ) | 10 | ( | 10.5% | ) |  |
|  | Unknown | 0 | ( | 0.0% | ) | 5 | ( | 5.3% | ) |  |
| Barthel Index | No disability | 279 | ( | 83.8% | ) | 54 | ( | 56.8% | ) | <0.001 |
|  | Moderate disability | 29 | ( | 8.7% | ) | 10 | ( | 10.5% | ) |  |
|  | Severe disability | 22 | ( | 6.6% | ) | 20 | ( | 21.1% | ) |  |
|  | Unknown | 3 | ( | 0.9% | ) | 11 | ( | 11.6% | ) |  |
| Pathology | UC | 315 | ( | 94.6% | ) | 94 | ( | 98.9% | ) | 0.089 |
|  | Non-UC | 18 | ( | 5.4% | ) | 1 | ( | 1.1% | ) |  |
| cT | Under 1 | 72 | ( | 21.6% | ) | 2 | ( | 2.1% | ) | <0.001 |
|  | 2 | 129 | ( | 38.7% | ) | 5 | ( | 5.3% | ) |  |
|  | Over 3 | 132 | ( | 39.6% | ) | 86 | ( | 90.5% | ) |  |
|  | Unknown | 0 | ( | 0.0% | ) | 2 | ( | 2.1% | ) |  |
| cN | 0 | 303 | ( | 91.0% | ) | 56 | ( | 58.9% | ) | <0.001 |
|  | 1 | 20 | ( | 6.0% | ) | 21 | ( | 22.1% | ) |  |
|  | 2-3 | 10 | ( | 3.0% | ) | 17 | ( | 17.9% | ) |  |
|  | Unknown | 0 | ( | 0.0% | ) | 1 | ( | 1.1% | ) |  |
| cM | 0 | 333 | ( | 100.0% | ) | 95 | ( | 100.0% | ) |  |
| Clinical stage | Under 1 | 72 | ( | 21.6% | ) | 0 | ( | 0.0% | ) | <0.001 |
|  | 2 | 123 | ( | 36.9% | ) | 0 | ( | 0.0% | ) |  |
|  | Over 3 | 138 | ( | 41.4% | ) | 95 | ( | 100.0% | ) |  |
| *UC* urothelial carcinoma |  |  |  |  |  |  |  |  |  |  |
