# Supplementary material for: Comparative Effectiveness of Radiation Versus Radical Cystectomy for Localized Muscle-Invasive Bladder Cancer
Source: Adv Radiat Oncol. 2022 Dec 27;8(3):101157. doi: 10.1016/j.adro.2022.101157 (PMC9991535; doi:10.1016/j.adro.2022.101157)
Supplement: Supplementary file 3 — Supplementary materials Supplementary material associated with this article can be found, in the online version, at XXX [file mmc3.docx]

| **Supplemental table2** Patient characteristics after propensity score matching between cystectomy group and radiation therapy (RT) group | | | | | | | | | | | | |
| --- | --- | --- | --- | --- | --- | --- | --- | --- | --- | --- | --- | --- |
|  |  |  |  |  |  |  |  |  |  |  | Standardized difference | |
|  |  | Cystectomy (n=77) | | | | RT (n=77) | | | | P-value | Pre-matching | Post-matching |
| Age: years median (range) |  | 73 | ( | 49-98 | ) | 75 | ( | 30-93 | ) | 0.486 | 0.428 | 0.050 |
| Sex | Male | 55 | ( | 71.4% | ) | 56 | ( | 72.7% | ) | 1.000 | 0.244 | 0.041 |
|  | Female | 22 | ( | 28.6% | ) | 21 | ( | 27.3% | ) |  |  |  |
| Charlson Comorbidity Index | 0 | 58 | ( | 75.3% | ) | 55 | ( | 71.4% | ) | 0.716 | 0.396 | 0.125 |
|  | Over 1 | 19 | ( | 24.7% | ) | 22 | ( | 28.6% | ) |  |  |  |
| Barthel Index | No disability | 67 | ( | 87.0% | ) | 63 | ( | 81.8% | ) | 0.601 | 0.393 | 0.203 |
|  | Moderate disability | 4 | ( | 5.2% | ) | 7 | ( | 9.1% | ) |  | 0.077 | 0.215 |
|  | Severe disability | 6 | ( | 7.8% | ) | 7 | ( | 9.1% | ) |  | 0.597 | 0.066 |
| Pathology | UC | 71 | ( | 92.2% | ) | 72 | ( | 93.5% | ) | 1.000 | 0.420 | 0.034 |
|  | Non-UC | 6 | ( | 7.8% | ) | 5 | ( | 6.5% | ) |  |  |  |
| cT | Under 2 | 40 | ( | 51.9% | ) | 39 | ( | 50.6% | ) | 1.000 | 0.502 | 0.037 |
|  | Over 3 | 37 | ( | 48.1% | ) | 38 | ( | 49.4% | ) |  |  |  |
| cN | 0 | 67 | ( | 87.0% | ) | 65 | ( | 84.4% | ) | 0.818 | 0.520 | 0.105 |
|  | 1-3 | 10 | ( | 13.0% | ) | 12 | ( | 15.6% | ) |  |  |  |
